# Implantation of Artificial*Iris*, a CustomFlex irisprosthesis, in a trauma patient with an Artisan lens: A case report and review

**DOI:** 10.1097/MD.0000000000008405

**Published:** 2017-11-10

**Authors:** Farideh Doroodgar, Mahmoud Jabbarvand, Feizollah Niazi, Sana Niazi, Azad Sanginabadi

**Affiliations:** a Farabi Eye Hospital, Tehran University of Medical Sciences; b Shahid Beheshti University of Medical Sciences and Health Services, Tehran, Iran.

**Keywords:** Artificial*Iris*, Artisan, customflex, trauma

## Abstract

**Purpose:**

To evaluate probable complications of Artificial*Iris* implantation with iris fixated intraocular lens.

**Method:**

Development of photophobia, glare, and psychological strain during face-to-face communication in a 23-year-old man with a widespread traumatic iris defect terminate to make a decision for performing implantation of an Artificial*Iris* (Humanoptics, Erlangen, Germany) under the remnant iris without removing the patient's existing Artisan lens.

**Results:**

Without any intraoperative or postoperative complications, the patient's visual acuity increased by 1 line, the endothelial cell loss was comparable with the cell loss associated with standard cataract surgery, and the anterior-chamber depth and anterior-chamber anatomy did not change. At the final follow-up examination, the mean intraocular pressure did not differ from baseline, and we achieved high level of patient satisfaction and subjective vision improvement. We discuss the particular importance of considering the patient's expectations, the appropriate measurements, ways to perfect color evaluation, and the types of Artificial*Iris* products.

**Conclusion:**

The implantation of the Artificial*Iris* in patients with aphakic iris-supported lenses (ie, pre-existing Artisan lenses) is a feasible approach and a useful option for patients with thin irises and iris hypoplasia who are at risk of subluxation or the dislocation of the posterior-chamber intraocular lens (PCIOL), and also those with sclerally fixed PCIOLs.

## 1 Introduction

Symptoms such as aberration disorders, contrast sensitivity restriction, dysphotopsia, depth of focus limitations, and ghosting phenomenon experiences (which can be remembered by AbCDefGh^∗^) can occur in eyes with normal irises; however, these adverse effects are particularly noticeable in patients with iris and pupil defects. The magnitude of higher-order aberrations (HOAs) is closely related to the pupil and pupil size, and HOAs are closely related to depth of focus.^[1,2]^ In addition to congenital aniridia, which can entail amblyopia,^[3]^ large iris defects and persistent mydriasis after ocular trauma are among the major indications for surgical interventions. Congenital diseases (eg, coloboma and aniridia), iatrogenic causes (eg, eye surgery in cases with intraoperative floppy iris syndrome [IFIS]),^[4]^ iris tumor excision, and iridocorneal endothelial syndrome (ICE)^[5]^ are less common causes. Many patients with large and multiple iris defects are not satisfied with conservative management techniques, such as sunglasses, tinted contact lenses,^[6]^ lamellar intrastromal corneal tattoos,^[7]^ or specific suture techniques^[8]^ (eg, iridoraphy or iridopexy side-to-side iris sutures). The Artificial*Iris* is foldable in its rolled state, and it can be inserted through a 3.0-mm incision. The previous generation of iris prostheses that have been used with and without penetrating keratoplasty^[9]^ are difficult to apply, can require large incisions, and might not have a realistic appearance.^[10]^ Few iris reconstruction studies have been published using the intended new type of silicon iris implant. This implant was developed in 1998 by Professor Dr Hans-Reinhard Koch and Dr Karlheinz Schmidt. Conformité Européenne approved Artificial*Iris* in 2011, and it is currently undergoing the US Food and Drug Administration approval process in the United States. This novel artificial iris is a handmade device for various surgical options.^[10–12]^ This implant is made of a foldable, highly biocompatible, and medical-grade silicone material. The anterior surface mimics the natural appearance of the iris with regard to its color composition (via embedded nontoxic pigments), and the iris structure is created from a Makrolon mold. The posterior surface, with its black pigmentation, completely prevents light transmission. Artificial*Iris* is designed without an optic to allow the surgeon to select the most appropriate intraocular lens (IOL) or optic for the patient. All Artificial*Iris* are 360°, 12.8-mm diameter disks with fixed pupils of 3.25 mm.^[13,14]^ Artificial*Iris* (Humanoptics, Erlangen, Germany) is known as the CustomFlex iris prosthesis in the United States. It comes in 2 types: an Artificial*Iris* with a suturable fiber with high mechanical stability preferred for partial implant surgery,^[12]^ and an Artificial*Iris* without fiber for easier handling and greater pliability in cases in which suturing is not indicated.^[12]^

To the best of our knowledge and based on a comprehensive literature search of PubMed, the ISI Web of Science, Google Scholar, and Scopus, no other studies exist on this topic, except 1 study of concomitant iris defects and Artisan lens implantation.^[15]^ This study is the first report of Artificial*Iris* (Humanoptics, Erlangen, Germany) implantation in a trauma patient with an existing Artisan lens. In this regard, traumatized eyes with highly diverse posttraumatic conditions might benefit from this device because of its outstanding outcomes. We present this case to report the results of this procedure.

## 2 Case report

**Figure 1:**
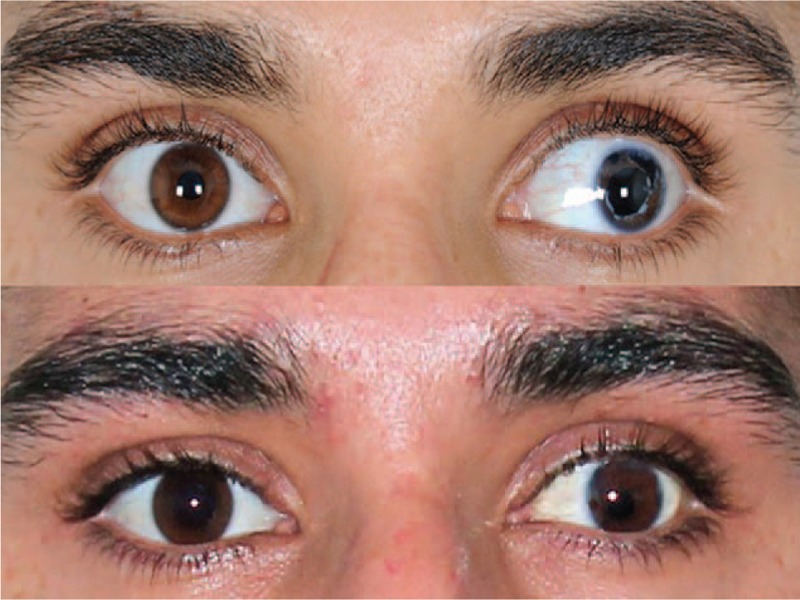
Photographs of the 23-year-old patient who sustained severe penetrating trauma of the left eye with a consecutive loss of the lens and iris and multiple eye surgeries, before (up) and after (down) artificial iris implantation (HumanOptics, Erlangen, Germany) without previous Artisan lens exchange. The pupil is well-centered and the color matches the fellow right eye. Even though the match between the 2 eyes may not be perfect in every case, from cocktail party distance, it is very difficult to see any difference between the 2 eyes.

**Figure 2:**
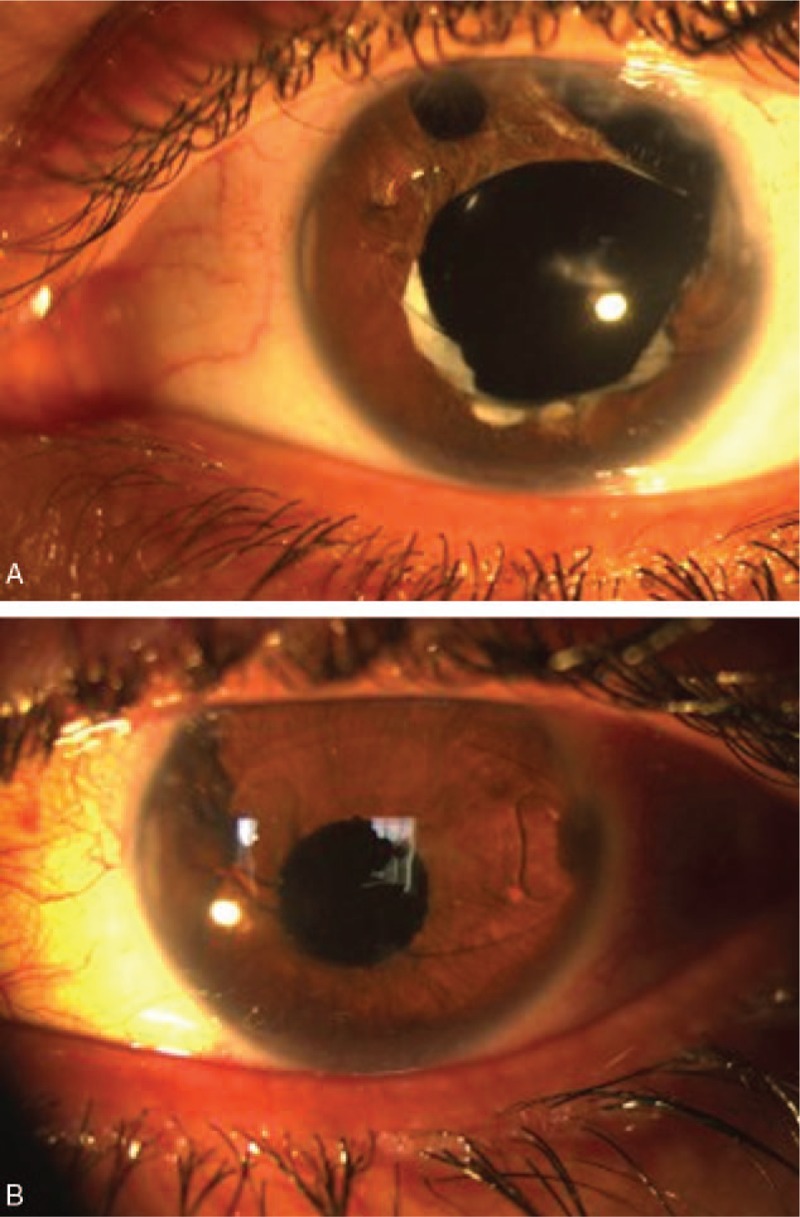
(A, B). Photo slit image before and after surgery.

In February 2014, a 23-year-old man presented with photophobia and a history of eye trauma (penetration) 10 years earlier. “I can’t communicate with my friends or participate in social activities because people stare at my disfigured eye,” he said (Fig. 1 and Fig. 2A, B). He was upset about his appearance and was unable to maintain eye contact comfortably, even with me. He was not satisfied with conservative management techniques (spectacles or contact lenses) or suturing the natural iris (via iridoraphy, iridopexy, or side-to-side iris sutures). The patient was advised to ignore his condition and focus on more positive things (exercise, listening to music, and other similar activities). The patient signed and received a copy of the written informed consent document that explained probable treatment complications, such as glaucoma, corneal decomposition, and consecutive surgeries. A customized Artificial*Iris* was ordered based on the patient's face, focusing on the color of his normal eye. In this case, an Artificial*Iris* with a fiber meshwork was ordered for its reliability. A second Artificial*Iris* was also requested as a backup.

The patient underwent anterior-segment reconstruction with the implantation of the Artificial*Iris* at the University Eye Hospital in 2016. The medical records were evaluated for changes in visual acuity as a functional parameter, for IOP values to assess secondary glaucoma, and for endothelial cell density (ECD).^[16]^ The angle grading upon gonioscopic examination^[17]^ was used as a quantitative parameter for the evaluation condition. Slit-lamp photography and anterior-segment optical coherence tomography (OCT)^[16]^ images were reviewed when available.

The candidate was examined before and after surgery when the wound healing was complete. Best-corrected visual acuity (BCVA) was assessed using a Snellen chart. IOP was measured using a standard Goldmann applanation tonometer and a Canon TX-10 noncontact tonometer (Canon USA Inc., One Canon Plaza, Lake Success, NY). The gonioscopic examination of the anterior-chamber angle (ACA) was performed in the dark using a Goldmann 3 mirror lens at a high magnification (X16), and all of the quadrants were graded in the primary position at 4:35°-45 using the Shaffer grading system.

White-to-white (W-W) was detected using calipers (Table 1) and an OrbscanIIZ (Bausch & Lomb, New York, NY). Sulcus-to-sulcus (S-S) distance was measured using an ultrasound biomicroscope (UBM Quantel Medical, Aviso S).^[18]^ Endothelial cell biomicroscopy was used to calculate ECD (CellChek XL; Canon Medical Inc., Irvine, CA).^[16]^ All of the parameters were assessed before surgery and also 1 day, 3 days, 1 week, 1 month, 2 months, 3 months, and monthly thereafter. The ACA characteristics were defined via spectral-domain OCT (SD-OCT) using a Cirrus OCT device (Carl Zeiss Meditec, Inc.) in an objective manner.^[17]^ The patient rated his satisfaction with the overall results on a scale from 1 to 10 (1 = none, 10 = maximum satisfaction).^[19]^

**Table 1:**
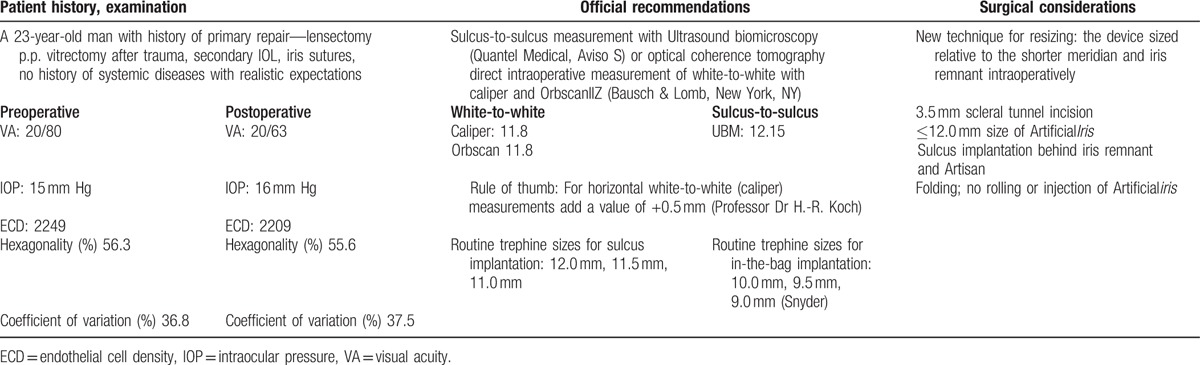
Patient characteristics and surgical considerations.

Surgery was performed using general anesthesia through a 3.5-mm scleral tunnel incision at the 12 o’clock position. Patient characteristics and surgical considerations are reported in the corresponding Table 1. The anterior chamber was filled with 1% sodium hyaluronate (Provisc), which was completely removed at the end of the surgery. Because the packaged Artificial*Iris* was 12.8 mm, cutting and resizing were performed to provide a ≤12-mm Artificial*Iris* with regard to the W-W and S-S preoperative evaluation and intraoperative eye measurement.

Some of the border was made slightly smaller (11 mm)^[20,21]^ than the measurements above for glaucoma prevention.

We cut and resized the Artificial*Iris* in additional places where the remnants of the iris formed a circular border. Support was provided for the Artificial*Iris* in the posterior segment, and the Artificial*Iris* was implanted under the remnant iris without removing the existing Artisan lens. Suturing of the device was not performed because of the sufficient support.

The superior and inferior leaflets were simultaneously unfolded using 2 hooks (folding was used in place of rolling or injection). We used bimanual instrumentation to ensure that endothelial contact was not made while another Neuhann chopper was placed through a second side port between the cornea and implant to prevent touching. Unfolding occurred posteriorly (not toward the endothelium). After implantation, the colored side faced up. The proper pupil centration of the Artificial*Iris* in the sulcus was evaluated via horizontal movements with the forceps. The centration was aesthetically pleasing, without any decentration or dislocation.

After the irrigation/aspiration of the anterior chamber and the stable positioning of the Artisan IOL was ensured, the scleral tunnel incision was sutured with nonabsorbable Nylon 10-0 thread. Postoperatively, 0.3% ciprofloxacin and 0.1% betamethasone eye drops were used 4 times per day for 1 week; then, ciprofloxacin use was discontinued, and betamethasone use was tapered during follow-up. To reduce the risk of postoperative inflammation, 1 mg/kg/d of oral steroids were used for the first 3 weeks and then tapered. During the follow-up period, the patient's visual acuity increased by 1 line, the anterior-segment depth and anterior-segment anatomy did not change significantly, the endothelial cell loss was comparable with the cell loss in standard cataract surgery, and the IOP increased to 25 mm Hg. The increase in IOP was only temporary and returned to normal during the follow-up examinations without medication. This case study shows the effectiveness of the small pupil in relieving the symptomatology associated with increased HOAs, including light sensitivity and ghosting. Furthermore, we achieved a high level of patient satisfaction and subjective vision improvement. The cosmesis empowered the patient, and he is eagerly looking forward to continuing his studies again.

We did not observe threatening endothelial damage (Table 1), retinal detachment, secondary glaucoma, bleeding, corneal edema, or dislocations associated with the device. Since the operation, the patient has contacted us in different ways, thanking us and claiming that this surgery was the best thing that has happened in his life: “I had not directly looked into people's eyes for years,” he said. Our colleagues even introduced us to 3 more patients who are currently being scheduled.

## 3 Discussion

To the best of our knowledge, this study is the first report of an Artificial*Iris* (Humanoptics, Erlangen, Germany) implantation in a trauma patient with an Artisan lens using a new method. Conformité Européenne approved use of Artificial*Iris* in 2011, and it is currently undergoing the US Food and Drug Administration approval process in the United States.^[16]^ The implantation of the Artificial*Iris*, particularly after iris-fixated IOL implantation with probable postoperative complications, such as intraocular inflammation, glaucoma,^[21]^ corneal edema, and endothelial cell loss,^[22]^ in traumatic cases, might provoke a lack of enthusiasm for these surgical procedures. Contradictory reports exist regarding certain cosmetic types^[23]^ of anterior-chamber Artificial*Iris* implantation in phakic eyes.^[24]^ In addition, some patients are unhappy with the necessary indications^[10]^ for aesthetic impairment (ie, AbCDefGh). Thus; we were motivated to make progress in this area of medicine. A recent case series concerning Artificial*Iris* reported high levels of patient satisfaction and postoperative vision improvement.^[10,13,16,21,25]^ The Artificial*Iris* should not be confused with other devices available under the trademark Newiris (Kahn Medical Devices Corp.) or other cosmetic implants.^[23]^

In our case, the visual acuity and anterior-segment depth did not change, the endothelial cell loss was comparable with the cell loss that occurs in standard cataract surgery, and the IOP increased to 25 mm Hg (which was only temporary and returned to normal during the follow-up examinations). We also achieved a high level of patient satisfaction and subjective vision improvement. In Mayer et al's prospective study, the mean anterior-chamber depth increased after combined cataract surgery and Artificial*Iris* implantation. This finding was related to the combined thickness of the Artificial*Iris*, the artificial lens, and the residual iris being less than that of the natural lens.^[16,18]^

The factors that contribute to the occurrence of glaucoma associated with Artificial*Iris* implantation might include patients with pre-existing glaucoma or a tendency toward glaucoma^[25]^ and techniques or materials that cause chronic irritation due to partially cut prostheses with mesh.^[21]^ Regular pre and postoperative measurements of IOP are required for Artificial*Iris* implantation. Because the trabecular meshwork plays an important role in aqueous outflow, the assessment of its anatomy in at-risk population might provide insight into 1 of the potential contributors to elevated IOP and the probability of glaucoma development. In this regard, although gonioscopic examination is the gold standard, it is a subjective procedure. For controversial cases, such as Artificial*Iris* implantation for patients (with or without pre-existing glaucoma) who are at risk for postoperative glaucoma,^[21,25]^ gonioscopy is useful, but in short supply. In contrast, objective evaluations might have better practical implications. The following methods might help and predict the size of the Artificial*Iris* with a better ACA definition: SD-OCT has a high sensitivity and low specificity for detecting angles compared with gonioscopy, and it does not require the placement of a scleral cup or corneal probe; ultrasound biomicroscopy (UBM) allows the investigation of the mechanisms that underlie angle closure; and the peripheral iris cannot be visualized via a Pentacam.^[16,17]^

Rickman considered hyperpigmentation of the iris remnant as a sign of the chronic irritation of the surrounding tissues via the sharp borders of the cut Artificial*Iris*.^[21]^ Using the full prosthesis without a mesh and a size smaller than originally planned is recommended to reduce the risk of complications, such as glaucoma.^[20,21]^ In this complex case of a perforating trauma with a corneal scar, an Artisan lens and a history of 9 surgeries (including primary repair, secondary IOL implantation, deep vitrectomies, and so on), a suturable Artificial*Iris* with fiber seemed more reliable than Artificial*Iris* without mesh.^[10,20,21,24,25]^ In contrast, to provide an appropriately sized (approximately 12-mm or less) Artificial*Iris* according to the OCT, W-W, and S-S preoperative evaluations^[16]^ and intraoperative measurements using a ruler,^[26]^ cutting and resizing are recommended because the Artificial*Iris* is sized at 12.8 mm. Even smaller (11 mm) irises have also been recommended^[20,21]^ for glaucoma prevention.

To achieve these goals, we resized the Artificial*Iris* where remnant iris was present. In other words, we sized the device relative to the shorter of the intraoperative meridians and gained better aesthetic results, which encouraged us to resize the Artificial*Iris* in the places with remnants with circular borders. This exclusive handmade device is friendly to surgeons’ hands, and it facilitates various surgical methods; the Artificial*Iris* can be suture fixated^[10,20]^ if necessary, sutured side-to-side to the remaining iris tissue,^[27]^ sutureless,^[14]^ or use knotless sutures.^[28]^ In eyes with remaining capsules, the Artificial*Iris* can be placed using the Rosenthal method in the sulcus or with a capsular tension ring (CTR)^[26]^ and staining (Trypan blue or indocyanine green in cases of congenital aniridia with a fragile capsule)^[29,30]^ in the bag. In these cases, no suturing is needed, and a more flexible variant of the Artificial*Iris* without the tissue layer can be used. As an alternative, we could have removed the patient's Artisan lens and stitched the new posterior-chamber IOL (PCIOL) to the scleral wall first; in the second step, the Artificial*Iris* would have been inserted on top of the IOL and fixated with sutures to achieve a 4-point fixation. Four-point fixation can be achieved using haptics with the Artificial*Iris* alone. The IOL could also have been sutured to the Artificial*Iris* first and then implanted together as a complex unit, necessitating a larger incision. These methods were not used because of the existence of sufficient support for the Artisan lens and the likely higher risk of complications during IOL exchange.

As is known, when combined with careful patient selection and the appropriate surgical technique, the posterior implantation of the Artificial*Iris* in aphakic and pseudophakic eyes can improve vision, the AbCDefGh^∗^ conditions, and positively affect quality of life while also providing satisfactory aesthetic results. Surgeons might sometimes be tempted to perform the seemingly convenient and less time-consuming method of implanting a foldable Artificial*Iris* via small-incision surgery; however, this method is not always an appropriate approach. In this report, we found that the implantation of the Artificial*Iris* in patients with aphakic iris-supported lenses (ie, pre-existing Artisan lenses) is a feasible approach and a useful option for patients with thin irises and iris hypoplasia who are at risk of subluxation or the dislocation of the PCIOL, and also those with sclerally fixed PCIOLs.

Patient expectations (ie, detailed informed consent explaining the risk of complications such as glaucoma and corneal decompensation), preoperative evaluations (objective and subjective), and postoperative examinations (EEE) are important to patient satisfaction, particularly in these specific cases.

In traumatic cases with unpredictable conditions, reperforming the intraoperative measurements and modifying the device based on the meridians might be possible. The surgeon might decide to use the Artificial*Iris*, most likely without mesh, with fewer complications; hence, having another type of Artificial*Iris* at hand as a backup adds to the convenience of the procedure and increases the likelihood of successful results.

## Acknowledgments

We acknowledge the professional manuscript services of American Journal Experts. We would like to acknowledge Dr Nima Jalali for their kind helps.
